# Effect of microplastics and watering regimes on a plant-soil system: Data on behavioural responses of an insect herbivore

**DOI:** 10.1016/j.dib.2021.107297

**Published:** 2021-08-14

**Authors:** Gabriele Rondoni, Elena Chierici, Alberto Agnelli, Eric Conti

**Affiliations:** a Department of Agricultural Food and Environmental Sciences, University of Perugia, Borgo XX giugno, 74, Perugia 06121, Italy; b Research Institute on Terrestrial Ecosystems (IRET-CNR), Via Madonna del Piano, 10, Sesto Fiorentino, 50019, Italy

**Keywords:** Behaviour, Fungus gnat, High-density polyethylene, Olfactometer, Sciaridae, Watering regime

## Abstract

The data presented here are related to the article titled “Microplastics alter behavioural responses of an insect herbivore to a plant-soil system” by Rondoni, G., Chierici, E., Agnelli, A., Conti, E. (2021). The data describe the changes in the attractiveness of a plant-soil system towards females of a herbivorous fungus gnat (Diptera: Sciaridae) when exposed to different combinations of the following treatments: (1) low or high plant (lentil)-soil watering regime; (2) absence (0%) or presence (5%) of HDPE microplastics in soil; (3) 1-day or 7-day duration of HDPE presence; (4) addition of fungus mycelium to the plant-soil system. We report data of female behaviour, i.e. the residence time in choice vs. no-choice sector of one-way olfactometers using a multiple olfactometer device.

## Specifications Table

**Table:** 

Subject	Agricultural and Biological Sciences (General)
Specific subject area	Effects of soil microplastics and watering regime on the behaviour of fungus-gnat females
Type of data	Table Image Figure
How data were acquired	J-Watcher, olfactometer, DNA extraction, DNA barcoding, SANGER sequencing; ImageJ
Data format	Raw Analyzed
Parameters for data collection	The behaviour (% residence time) of fungus gnat mated females was evaluated for 10 min in a multiple one-way olfactometer device at 25 °C. Odour sources (i.e., single pots of a plant-soil system, a fungus colony or clean air) were placed in glass chambers sealed with parafilm to a Teflon disk. A humified airflow conveyed the volatiles from the chamber to each olfactometer. DNA from insect tissues was amplified with universal primers for Sanger sequencing. For species identification, Sanger sequences were compared to sequences deposited in GenBank using BLAST.
Description of data collection	J-Watcher recordings of residence time data; ImageJ for description of plastic particle sizes. DNA barcoding (Sanger sequencing) for insect identification.
Data source location	Dipartimento di Scienze Agrarie, Alimentari ed Ambientali, Università degli Studi di Perugia, Perugia, Italy
Data accessibility	With the article plus DNA sequence in NCBI (accession numbers: MW947257-MW947259 available at https://www.ncbi.nlm.nih.gov/nuccore?term=((MW947257.1%5BAccession%5D)%20OR%20MW947258.1%5BAccession%5D)%20OR%20MW947259.1)
Related research article	[1] Rondoni, G., Chierici, E., Agnelli, A., Conti, E., Microplastics alter behavioural responses of an insect herbivore to a plant-soil system, Science of the Total Environment, 787: 147716. Doi: 10.1016/j.scitotenv.2021.147716

## Value of the Data

• These data provide a useful insight in the behavioural response of a fungus gnat herbivore insect towards a plant-soil system exposed to a combination of microplastic pollution and different watering regimes.
• These data can be useful for researchers focusing on insect behaviour and on the influence of pollutants, such as HDPE microplastics.
• These data can be compared to future studies to reveal the impact of microplastics on plant soil systems on herbivorous insects.

## 1 Data Description

**Fig. 1:**
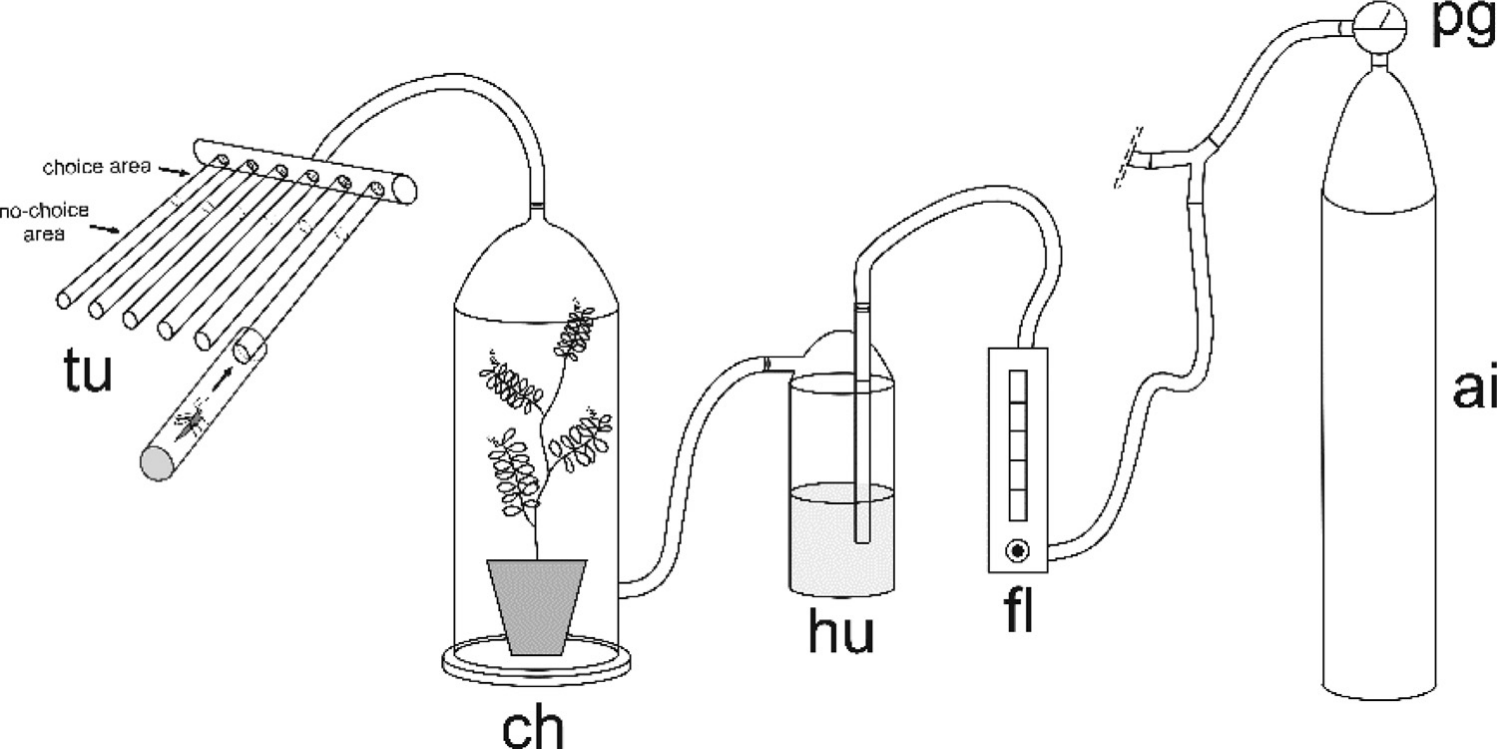
**Schematic of the one-way multiple olfactometer device (only one of two identical parts originating from a single air tank is shown).** “ai” = pressurized medical-grade air tank; “pg” = pressure gauge; “fl” = flowmeter; “hu” = humidifier; “ch” = glass chamber containing the odour source; “tu” = glass tube olfactometers independently connected to the airflow source and where the insects were allowed to move freely.
(2) Box-plot of fungus gnat residence time under different watering regimes of plant-soil system (Fig. 2). Box-plot of residence time (% of time spent in the choice area) of females when exposed to odours from different experimental treatments in one-way olfactometer. AIR: clean air; LW: low-watered plants; HW: high-watered plants.

The data underpin the research article entitled “Microplastics alter behavioural responses of an insect herbivore to a plant-soil system”, by Rondoni, G., Chierici, E., Agnelli, A., Conti, E.  [1]. The data include:

(1) Schematic of the one-way multiple olfactometer device where the insects were allowed to move freely (Fig. 1). Only one of two identical parts originating from a single air tank is shown in the figure.

**Fig. 2:**
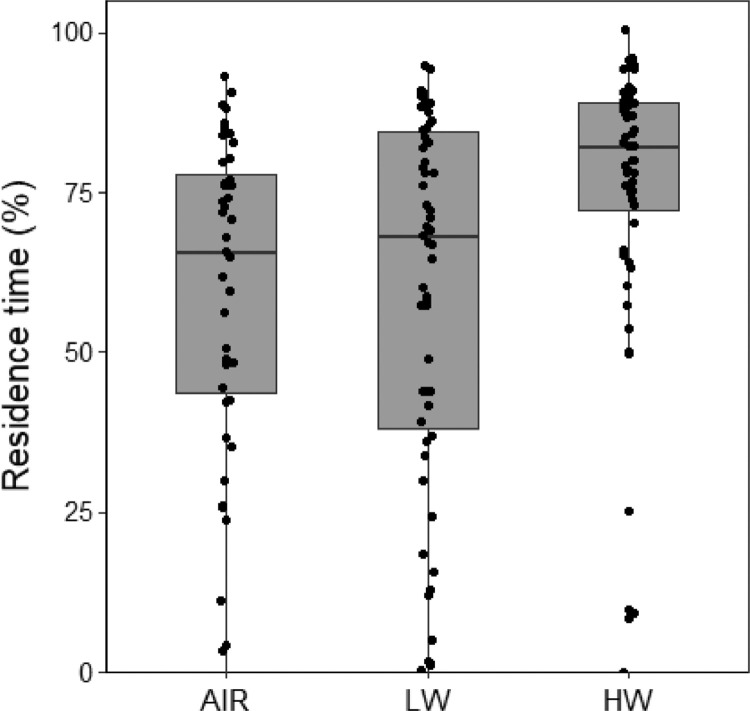
**Effect of watering regime on** fungus gnat females**.** Box-plot of residence time (% of time spent in the choice area) of females when exposed to odours from different experimental treatments in one-way olfactometer. AIR: clean air; LW: low-watered plants; HW: high-watered plants.
(3) Box-plot of fungus gnat residence time when exposed to plants without or with HDPE in soil (Fig. 3). Box-plot of residence time (% of time spent in the choice area) of females when exposed to odours from different experimental treatments in one-way olfactometer. 1d: 1-day duration of plant treatments; 7d: 7-day duration of treatments; 0%HDPE: no microplastics in soil; 5%HDPE: 5% concentration of microplastics in soil.

**Fig. 3:**
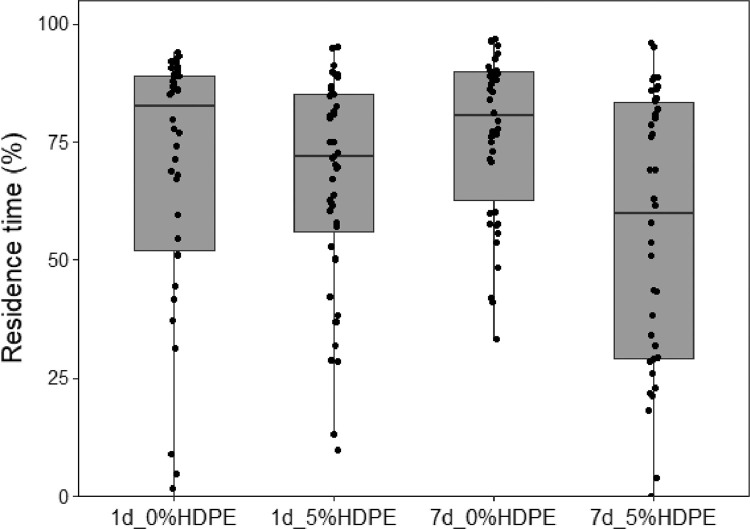
**Effect of microplastics on** fungus gnat females**.** Box-plot of residence time (% of time spent in the choice area) of females when exposed to odours from different experimental treatments in one-way olfactometer. 1d: 1-day duration of plant treatments; 7d: 7-day duration of treatments; 0%HDPE: no microplastics in soil; 5%HDPE: 5% concentration of microplastics in soil.
(4) Box-plot of fungus gnat residence time under combinations of watering regimes and microplastics (Fig. 4). Box-plot of residence time (% of time spent in the choice area) of females when exposed to odours from different experimental treatments in one-way olfactometer. 0%HDPE: no microplastics in soil; 5%HDPE: 5% concentration of microplastics in soil; LW: low-watered plants; HW: high-watered plants.

**Fig. 4:**
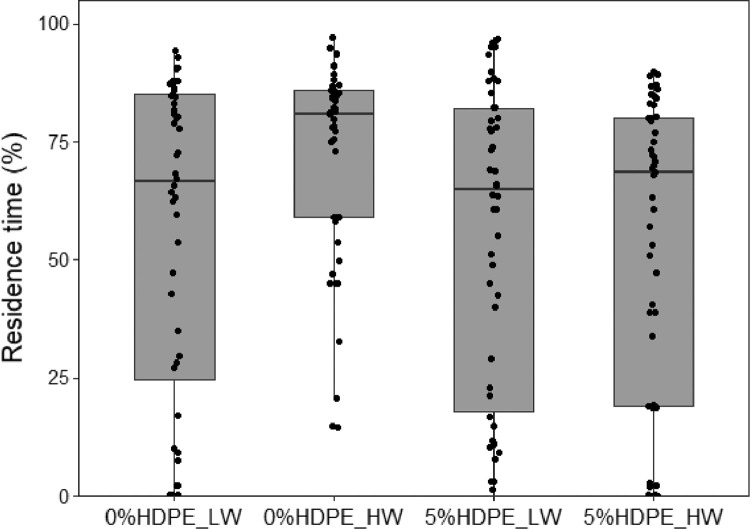
**Combined effect of watering regime and microplastics on** fungus gnat females**.** Box-plot of residence time (% of time spent in the choice area) of females when exposed to odours from different experimental treatments in one-way olfactometer. 0%HDPE: no microplastics in soil; 5%HDPE: 5% concentration of microplastics in soil; LW: low-watered plants; HW: high-watered plants.
(5) Box-plot of fungus gnat residence time under combinations of microplastics and *Fusarium proliferatum* (Fig. 5). Box-plot of residence time (% of time spent in the choice area) of females when exposed to odours from different experimental treatments in one-way olfactometer. Fus: *F. proliferatum*; 0%HDPE+Fus: plants subjected to high watering regime, no microplastics in soil and F. proliferatum; 5%HDPE: plants subjected to high watering regime, 5% concentration of microplastics in soil and *F. proliferatum*.

**Fig. 5:**
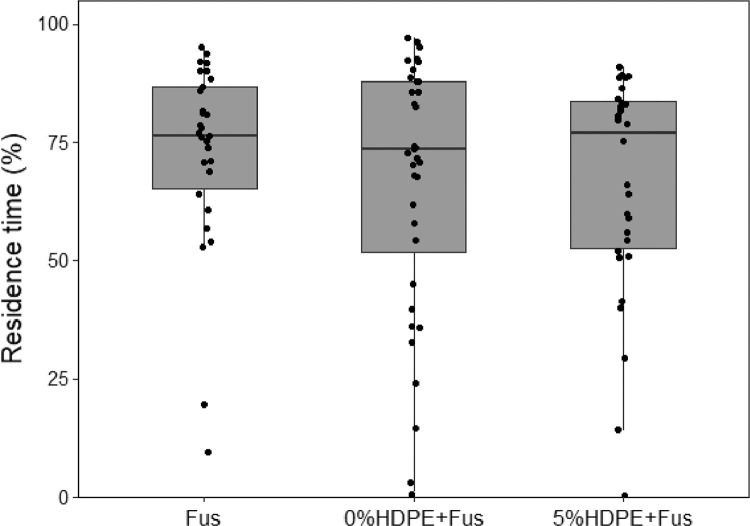
**Combined effect of *Fusarium proliferatum* and microplastics on** fungus gnat females**.** Box-plot of residence time (% of time spent in the choice area) of females when exposed to odours from different experimental treatments in one-way olfactometer. Fus: *F. proliferatum*; 0%HDPE+Fus: plants subjected to high watering regime, no microplastics in soil and *F. proliferatum*; 5%HDPE: plants subjected to high watering regime, 5% concentration of microplastics in soil and *F. proliferatum*.
(6) Chemical and physical parameters of the universal horticultural substrate (Gaia®) used in the bioassays (Table 1).

**Table 1:** Chemical and physical parameters of the soil substrate used for the bioassays.

**Basic cultivation substrates**
pH 6.5
Electrical conductibility 0.5 dS/m
Dry bulk density 160 kg/m^3^
Total porosity % of volume (v/v) 92% v/v
Components: peat (acidic), simple matrix non-composted (coir fiber and pitch), green compost
Nitric Nitrogen- N-NO3: 15.30 mg/l
Ammonia Nitrogen- N-NH4: 51.81 mg/l
organic substance: 74.55 dry substance
Cobalt: <0.5 µg/l
Molybdenum: 6 mg/kg dry substance
Cadmium: <0.1 mg/kg dry substance
Copper: 3.6 mg/kg dry substance
Nickel: <1 mg/kg dry substance
Lead: <1 mg/kg dry substance
Zinc: 24 mg/kg dry substance
Mercury: <0.05 mg/kg dry substance
Chromium: <1 mg/kg dry substance
Arsenic: <0.1 mg/kg dry substance
**Raw material**
Blond peat: 0-25 mm size
Brown peat: 0-25 mm size
Coir fiber: fine fraction
Compound inorganic fertilizer NPK.
Standard methods: pH (EN 13037), EC (EN 1313038), Dry bulk density, Total porosity (EN 13041),
commercial volume (EN 12580)
National reference: legislative decree N° 75 of 29 April 2010-Annex 4, Basic cultivation substrates
**Physical properties**
Apparent density: 270–300 g/l
Air volume: 19–23 %
Water retention: 5.3 g/g
Capillary ascent: 25 %
Structure: medium-fine
**Chemical properties**
E.C. mS/cm (Sonneveld method 1:1,5 v/v): <1.5
E.C. mS/m (EN 13038method 1:5 v/v): ∼ 55.00
**Company:** GEOTEC S.r.l. Via Copernico Nr 11- I-39100 Bolzano (Italy)
**Production:** Cavanella Po- Adria (RO) (Italy)
(7) Raw data (residence time, i.e., the % of time spent in the choice area) for Fig. 2, Fig. 3, Fig. 4, Fig. 5 (Supplementary Materials).

## 2 Experimental Design, Materials and Methods

Chips (2 cm each) of High-Density Polyethylene (HDPE) were provided by Rigenera s.r.l. (Terni, 131 Italy). Using an electric hammer mill (M13, Omas s.r.l., Padova, Italy), fragments were progressively ground in small particles. HDPE particles smaller than 0.25 mm (average: 157 µm 135 ± 4.3 SE) were used for subsequent bioassays. Pictures of the HDPE particles were taken under a stereomicroscope. Then, particle size was measured using ImageJ [2,3].

The herbivore species under investigation was *Bradysia difformis* Frey, 1948, junior synonym to *Bradysia impatiens* (Johannsen, 1912) [4,5]. Other synonyms are: *Bradysia paupera* Tuomikoski, 1960 and *Bradysia agrestis* Sasakawa, 1978. This species is a worldwide pest of economically important crops and ornamental plants [6,7]. Fungus gnat identification was conducted using DNA barcoding from insect legs [8]. Total DNA was purified from insect legs of 4 specimens using QIAGEN DNeasy Blood & Tissue Kit (QIAGEN Inc., Chatsworth, CA, USA). Successful extraction was assessed through 1% agarose gel run. A Cytochrome c oxidase subunit I was amplified in PCR reactions using the universal primers LCO1490 and HCO2198 [9]. Reaction products were run in an agarose gel (1.5%). Only the reactions that exhibited one clear amplicon band of the expected length were sent to sequencing. Each amplicon was sequenced in both forward and reverse directions by BMR Genomics (Padova, Italy). Consensus sequences were compared to sequences deposited in GenBank using BLAST [10]. Representative sequences were deposited in NCBI (accession numbers: MW947257-MW947259).

Bioassays were performed daily from 9:00 to 14:00 at 25 °C. A multiple olfactometer device (Fig. 1), composed of 12 single one-way olfactometers, was designed to allow the simultaneous behavioural detection of 12 fungus gnat females. Olfactometer has been demonstrated to adequately describe adult fungus gnat behaviour [11]. For the bioassays with plants, groups of 20 *Lens culinaris* Medik. were grown in plastic pots containing Gaia® universal horticultural substrate (details in Table 1). For all the bioassays, females were isolated in a small tube (40 mm length, 10 mm diameter) 30 min before the bioassay and transferred to the bioassay room for acclimatization [12]. Four different experiments were evaluated:

(1) high watering (HW) plant-soil system provided with 20 mL of tap water every day versus low watering (LW) plant-soil pots irrigated every 2 days with 5 mL of tap water (Fig. 2);
(2) plants without HDPE in soil (0%) versus plants with HDPE (5%), both tested after 1 day or 7 days under high watering regime (Fig. 3);
(3) combined effect of HW versus LW watering regime in absence (0%) or presence of soil microplastics (5%). Attractiveness of plant-soil systems were evaluated after 7 days from the transplanting (Fig. 4);
(4) a colony of the fungus *Fusarium proliferatum* (Matsushima) versus a colony of the fungus positioned along with plants subjected to an HW regime with 5% and without HDPE (Fig. 5).

The position (choice vs. no-choice zone) of each female in the olfactometer tube was tracked by two operators and recorded using J-Watcher [13]. The residence time, i.e., the time spent by a female in the choice zone (i.e., the time spent in the olfactometer sector closer to the stimulus) of the olfactometer, was calculated over the total time of the bioassay (600 s). Residence time data were subjected to arcsine square root transformation before analysis. Thirty to 60 insects were bioassayed for each experiment. Three to 6 repetitions with the same stimulus were conducted. For each of the four experiments, the effect of treatment was evaluated with linear models followed by multiple comparisons procedure (significance at *P* ≤ 0.05). The effect of blocks (insects that were screened against the same stimulus) was initially included as a random effect in generalized mixed models and evaluated with likelihood ratio test, but its relevance was never justified [14,15].

## CRediT Author Statement

**Gabriele Rondoni:** Conceptualization, Methodology, Formal analysis, Investigation, Resources, Writing – original draft, Writing – review & editing, Funding acquisition; **Elena Chierici:** Investigation, Resources, Writing – original draft, Writing – review & editing; **Alberto Agnelli:** Resources, Writing – review & editing, Funding acquisition; **Eric Conti:** Conceptualization, Methodology, Resources, Writing – original draft, Writing – review & editing, Funding acquisition.

## Declaration of Competing Interest

The authors declare that they have no known competing financial interests or personal relationships which have or could be perceived to have influenced the work reported in this article.

## Appendix Supplementary materials

Image, application 1

## References

[1] Rondoni G., Chierici E., Agnelli A., Conti E. (2021). Microplastics alter behavioural responses of an insect herbivore to a plant-soil system. Sci. Total Environ..

[2] Rasband W.S. (1997). Image J. US National Institutes of Health.

[3] Rondoni G., Bertoldi V., Malek R., Djelouah K., Moretti C., Buonaurio R., Conti E. (2018). Vicia faba plants respond to oviposition by invasive *Halyomorpha halys* activating direct defences against offspring. J. Pest Sci..

[4] Mohrig W., Heller K., Hippa H., Vilkamaa P., Menzel F. (2013). Revision of the black fungus gnats (Diptera: Sciaridae) of North America. Stud. Dipterol..

[5] Ye L., Leng R., Huang J., Qu C., Wu H. (2017). Review of three black fungus gnat species (Diptera: Sciaridae) from greenhouses in China: three greenhouse sciarids from China. J. Asia Pac. Entomol..

[6] Cloyd R.A. (2015). Ecology of fungus gnats (*Bradysia* spp.) in greenhouse production systems associated with disease-interactions and alternative management strategies. Insects.

[7] Wiraguna E., Malik A.I., Erskine W. (2017). Waterlogging tolerance in lentil (*Lens culinaris* Medik. subsp. *culinaris*) germplasm associated with geographic origin. Genet. Resour. Crop Evol..

[8] Rondoni G., Fenjan S., Bertoldi V., Ielo F., Djelouah K., Moretti C., Buonaurio R., Ricci C., Conti E. (2018). Molecular detection of field predation among larvae of two ladybird beetles is partially predicted from laboratory experiments. Sci. Rep..

[9] Folmer O., Black M., Hoeh W., Lutz R., Vrijenhoek R. (1994). DNA primers for amplification of mitochondrial cytochrome c oxidase subunit I from diverse metazoan invertebrates. Mol. Mar. Biol. Biotechnol..

[10] Massaccesi L., Rondoni G., Tosti G., Conti E., Guiducci M., Agnelli A. (2020). Soil functions are affected by transition from conventional to organic mulch-based cropping system. Appl. Soil Ecol..

[11] Cloonan K.R., Andreadis S.S., Chen H., Jenkins N.E., Baker T.C. (2016). Attraction, oviposition and larval survival of the fungus gnat, *Lycoriella ingenua*, on fungal species isolated from adults, larvae, and mushroom compost. PLoS One.

[12] Rondoni G., Bertoldi V., Malek R., Foti M.C., Peri E., Maistrello L., Haye T., Conti E. (2017). Native egg parasitoids recorded from the invasive *Halyomorpha halys* successfully exploit volatiles emitted by the plant-herbivore complex. J. Pest Sci..

[13] Bertoldi V., Rondoni G., Brodeur J., Conti E. (2019). An egg parasitoid efficiently exploits cues from a coevolved host but not those from a novel host. Front. Physiol..

[14] Zuur A.F., Ieno E.N., Walker N.J., Saveliev A.A., Smith G.M. (2009). Mixed Effects Models and Extensions in Ecology with R.

[15] Rondoni G., Ielo F., Ricci C., Conti E. (2017). Behavioural and physiological responses to prey-related cues reflect higher competitiveness of invasive vs. native ladybirds. Sci. Rep..

